# A new Editor-in-chief for *Synthetic Biology*

**DOI:** 10.1093/synbio/ysae006

**Published:** 2024-04-16

**Authors:** Sonja Billerbeck

**Affiliations:** Molecular Microbiology, Groningen Biomolecular Sciences and Biotechnology Institute, University of Groningen, Groningen 9747 AG, The Netherlands

In January of this year, I took over the position of Editor-in-Chief (EIC) for *Synthetic Biology* from the founding EiC, Jean Peccoud.

It is a real privilege to serve in this role for the next few years. I am looking forward to the opportunities, but I am aware of the responsibility that comes with an editorial leadership position. Academic journals play a pivotal role as stewards of scientific integrity and as platforms for shaping the discourse on future advancements in the field. Journals also have the potential to influence scientific culture and amplify the voices of diverse participants within the scientific community.

**Figure 1.**
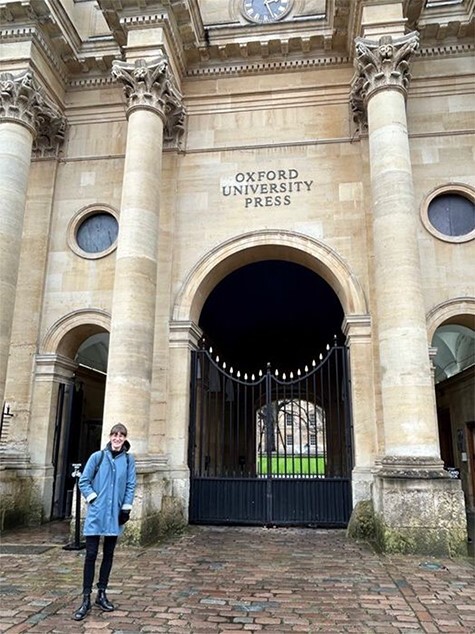
A recent visit to the headquarters of Oxford University Press (OUP) in Oxford, UK. OUP is the publishing house of University of Oxford, one of the largest university press houses in the world.

In this editorial, I introduce myself and highlight the journal’s aims, scope and future focus areas. Figure 1 shows me during a recent visit at Oxford University Press in Oxford, UK, where I discussed this new role with the editorial office.

In my daily life, I am a faculty member at the University of Groningen, located in the Northern Netherlands. My research expertise ranges from protein engineering to the engineering of microbial systems for applications in health and sustainability. My professional journey has taken me from Germany to Switzerland, the USA and the Netherlands, fostering a robust network within the global synthetic biology community. Furthermore, for more than a decade, I have been involved in the synthetic biology student competition iGEM.

I first engaged with the journal several years ago as a frequent author of News articles, which are commissioned 500-word pieces that communicate recent developments in synthetic biology to a broad audience. A role that I found intellectually stimulating as it allowed me to distill and contextualize recent advancements in *Synthetic Biology*. This experience led to my subsequent roles as an Associate Editor and News Editor for the Journal.

*Synthetic Biology* was founded seven years ago, covering all subject areas of synthetic biology, including engineered prokaryotic, fungal, archaeal, mammalian and plant systems, mathematical modeling, bioinformatics, educational and societal aspects. In addition to the classical research articles, the journal publishes well-curated datasets and advances in *Synthetic Biology* education.

The journal has released a few special issues (SIs) highlighting subjects of interest to the community such as synthetic biology education or biofoundries (1). One of my favorites is the recent SI on reproducibility, a first-of-its-kind collection that shines a light on reproducibility in synthetic biology through current work in the space and views on what is needed to move forward (2).

Looking ahead, I envision the continuity of the journal’s core aims and scope alongside a strategic push for heightened global visibility.

My focal point will be on early-career researchers and creating opportunities to involve them in the field of synthetic biology. I find it important to encourage and enable diverse young researchers to take charge of their field’s future and have chances to shape it themselves.

First, I invite early-career researchers with academic credentials (PhD-level) and publishing experience to actively reach out to our editorial office in order to get included in our reviewer database (3).

Second, we invite early-career scholars with a keen interest in writing to contribute to our News article collection by reaching out to the journal’s News Editor or myself.

Third, you can follow our social media channels, where I announce recently published articles and conferences in the field where I can be met in person to discuss research and potential submissions (4).

I look forward to interacting with the synthetic biology community and meeting some of you in person.
